# Genetic Advances in the Study of Speech and Language Disorders

**DOI:** 10.1016/j.neuron.2010.10.001

**Published:** 2010-10-21

**Authors:** D.F. Newbury, A.P. Monaco

**Affiliations:** 1 Wellcome Trust Centre for Human Genetics, Roosevelt Drive, Headington, Oxford OX3 7BN, UK

## Abstract

Developmental speech and language disorders cover a wide range of childhood conditions with overlapping but heterogeneous phenotypes and underlying etiologies. This characteristic heterogeneity hinders accurate diagnosis, can complicate treatment strategies, and causes difficulties in the identification of causal factors. Nonetheless, over the last decade, genetic variants have been identified that may predispose certain individuals to different aspects of speech and language difficulties. In this review, we summarize advances in the genetic investigation of stuttering, speech-sound disorder (SSD), specific language impairment (SLI), and developmental verbal dyspraxia (DVD). We discuss how the identification and study of specific genes and pathways, including *FOXP2*, *CNTNAP2*, *ATP2C2*, *CMIP*, and lysosomal enzymes, may advance our understanding of the etiology of speech and language disorders and enable us to better understand the relationships between the different forms of impairment across the spectrum.

## Main Text

### Speech and Language Disorders

Developmental disorders of communication represent one of the most common reasons for pediatric referrals (Harel et al., 1996) and account for a large proportion of statements of educational need (Law et al., 2000). This clinical category includes many individuals in whom speech and language problems are symptomatic of a more global developmental condition, such as autistic spectrum disorder, learning disability, or hearing impairment. For others, the speech and language deficit occurs in an otherwise normal developmental trajectory and has no obvious cause (Bishop, 1994). Such primary speech and language disorders are currently classified into five distinct categories: expressive language disorder, mixed receptive-expressive language disorder, phonological disorder, stuttering, and communication disorder not-otherwise-specified (DSM-IV). Disorders of speech involve an impairment in the production of fluent and comprehensible speech and include stuttering, in which the fluency of speech is disrupted; phonological disorder (including speech-sound disorder [SSD]), in which the problem lies in the production and proper use of speech sounds; and developmental verbal dyspraxia, in which there is impairment in the coordination and motor control of the speech organs. Disorders of language are perhaps less perceptible but no less profound. They may involve problems with the correct formation of words (morphology) or sentences (syntax), the derivation of meaning (semantics), or the use of linguistic context (pragmatics) and may affect expressive and/or receptive language as well as nonverbal language (e.g., reading and writing—developmental dyslexia). The term specific language impairment (SLI) is often used as an umbrella term for expressive language disorder, mixed receptive-expressive language disorder, and sometimes phonological disorder.

Although many of these speech and language disorders are differentiated at a clinical level, they are highly comorbid with each other. Many children do not fall neatly into a single diagnostic cluster, and others may change between categories as their language develops. Approximately 15% of children with persistent speech disorders also have a language disorder and approximately 5% of children with SLI also experience speech difficulties (Shriberg et al., 1999). Over 60% of stutterers present with concurrent speech and language disorders, the most common of which is articulation disorder (Blood et al., 2003). Furthermore, although the diagnostic criteria for SLI necessitates the absence of explanatory medical conditions, studies have found that affected individuals are at an increased risk of associated developmental delays, cognitive impairment, social problems, literacy deficits, and behavioral difficulties (Conti-Ramsden and Botting, 1999; Conti-Ramsden et al., 2001; Law et al., 2000; Wadman et al., 2008). The exact relationship between speech and language deficits and these other developmental problems remains a matter of debate, and the etiological basis of these overlaps are unclear.

It is well documented that genetic factors contribute to susceptibility to speech and language impairments. Speech and language deficits are heritable and show strong familial aggregation (e.g., Barry et al., 2007; Clark et al., 2007; Conti-Ramsden et al., 2007; Lewis et al., 2007). Moreover, twin studies report an increase in monozygotic twin concordance rates over that of dizygotic twins, suggesting that much of this aggregation can be attributed to genetic influences (Bishop, 2002; Felsenfeld et al., 2000; Hayiou-Thomas, 2008). Nonetheless, it is generally thought that the genetic mechanisms underlying susceptibility to speech and language disorders are multifactorial in nature, involving complex interactions between several common genetic variants and environmental factors. Despite this complexity, researchers have recently begun to identify genetic factors that may play a role in the etiology of speech and language disorders (Kang et al., 2010; Newbury et al., 2009; Vernes et al., 2008). It is hoped that the identification of contributory genetic risk factors will allow the elucidation of biological pathways and neurological mechanisms that contribute to speech and language acquisition processes and play a critical role in the etiology of speech and language disorders. In turn, the investigation of identified genetic factors may help untangle the complex relationships between speech and language disorders and related developmental conditions.

In this review, we provide an overview of recent developments in the genetic study of spoken developmental speech and language disorders. We do not describe the study of developmental dyslexia as excellent reviews of this area have recently been published (Scerri and Schulte-Körne, 2010).

### Methods for Identifying Contributory Genetic Variants

For some genetic disorders, it is possible to select candidate genes on the basis of their function alone. However, for speech and language disorders, in which the underlying biological mechanism is unclear, the identification of susceptibility genes usually starts with an unbiased screening approach. This step allows the identification of a candidate region and thus acts to reduce the number of possible contributory genes to a manageable size prior to a more in-depth investigation. These screening approaches usually take the form of genome-wide linkage or association studies (Elston and Anne Spence, 2006).

In a linkage study, one investigates families with members affected by the disorder under study. By genotyping polymorphic genetic markers spread across the genome, it is possible to generate measures of genetic identity between sibling pairs. Linkage studies look for regions of the human genome in which there is a correlation between the level of genetic identity and the level of phenotypic similarity for any given sib pair. Perhaps the most simple example of linkage analysis is provided by the study of single-gene disorders in large families. In such circumstances, one is able to identify specific chromosome segments that cosegregate with disease status between affected relative pairs. Nonetheless, as discussed below, the study of such pedigrees in complex disorders is the exception rather than the rule. Instead, the majority of linkage studies for complex disorders investigate large numbers of smaller nuclear families and compare genetic identity between sibling pairs within family units. Such analyses can be performed under the assumption of a defined genetic model (parametric linkage analysis) or by using a model-free method (nonparametric linkage analysis). Linkage analyses provide a robust and powerful method of detecting contributing genetic factors but afford a low level of resolution. Thus, although linkage studies can be completed with relatively small sample sizes, they often lead to the identification of large genetic intervals, which necessitate follow-up higher-resolution (targeted association) investigations to allow the identification of a specific candidate gene. Linkage studies are usually completed at a genome-wide level (i.e., across all chromosomes—i.e., a genome-wide linkage analysis or GWLA) but may target specific chromosome regions (targeted linkage) if the researcher has an a priori reason to do so.

Because linkage studies simply investigate the level of genetic sharing (joint inheritance), they do not allow the identification of specific contributory genetic variants. For this crucial step, an association study is required. In contrast to linkage, an association study is based upon the principle that contributory genetic variants will be more common in affected (cases) than unaffected (controls) individuals. In the simplest example, if a given genetic variant was necessary and sufficient for the onset of the disorder then we would expect this variant to be present in 100% of cases but never in controls. In complex disorders, the effect is not expected to be so stark but the rationale still holds. Association studies provide a better resolution of genetic information than linkage studies but are not as powerful and necessitate the genotyping of a high number of genetic markers in extremely large samples. These factors bring their own complications in terms of the logistics of data generation and multiple testing issues. Nonetheless, it is now possible to routinely generate genotypes for 2.5 million genetic variants (single-nucleotide polymorphisms [SNPs]) in thousands of case and control individuals enabling a so-called genome-wide association (GWA) study. The fact that association models were developed for case-control cohorts means that they often do not provide a convenient method for the fine mapping of linkage peaks in family-based samples. However, it is possible to apply such methods to genetic data collected from families by using parents or unaffected siblings as controls (Lunetta et al., 2000) or by applying quasi-association extensions (Abecasis et al., 2000). Such methods provide an important solution to fine mapping and the investigation of quantitative traits and may offer increased power to detect etiological variants.

It should be noted that although association studies are theoretically capable of identifying specific causal variants with relatively low effect sizes, in practice the genetic variants identified by association studies are not necessarily functional. Instead, they tend to be proxies that mark the approximate position of the etiologic genetic variant within a small segment of DNA. Thus, while association studies provide a strong starting point for functional studies, even high-density SNP association screens will require follow-up investigations to enable proof of causality (McCarthy and Hirschhorn, 2008).

Linkage and association investigations of speech and language disorders followed on from similar investigations of the related disorder developmental dyslexia in the 1990s (Scerri and Schulte-Körne, 2010). Over the last decade, researchers of speech-sound disorder have applied targeted linkage studies, while investigators of SLI and stuttering have performed genome-wide linkage studies and subsequent targeted association studies. In addition, a rare and specific form of verbal dyspraxia has been attributed to mutations of a specific gene known as *FOXP2*. A summary of loci that have been implicated in these various speech and language disorders, as well as particular genes that have been identified, is provided in Table 1.

#### FOXP2

The first gene to be implicated in a speech and language disorder was identified by the investigation of a large family affected by a distinctive form of speech impairment known as verbal dyspraxia. Verbal dyspraxia is characterized by difficulties in the control of orofacial muscles leading to a deficit in the production of fluent speech. In addition to their speech problems, affected members of this family also had expressive and receptive language deficits and, in some cases, written language problems and nonverbal cognitive impairment (Watkins et al., 2002). The remarkable thing about the speech and language impairments observed in this family was that the pattern of inheritance indicated that they may be caused by a mutation in a single gene (Vargha-Khadem et al., 1995). Genome-wide linkage analysis identified linkage to chromosome 7q (Fisher et al., 1998) and fine mapping of the locus indeed identified a mutation in the *FOXP2* gene (OMIM 605317) that was present in all affected family members but not in unaffected individuals (Lai et al., 2001). The relevance of *FOXP2* mutations to other cases of verbal dyspraxia was supported by the identification of an unrelated child with a similar form of speech impairment who was found to have a chromosome rearrangement that disrupted the *FOXP2* gene (Lai et al., 2001). These data have since been confirmed by several independent *FOXP2* screening studies that have identified additional individuals with disruptions of this gene, all of whom have syndromes that feature verbal dyspraxia (Feuk et al., 2006; Lai et al., 2000; Lennon et al., 2007; MacDermot et al., 2005; Pariani et al., 2009; Shriberg et al., 2006; Tomblin et al., 2009; Zeesman et al., 2006). The *FOXP2* gene encodes a winged helix/forkhead DNA-binding protein from the FOX family. This protein acts as a transcriptional repressor and has four alternative isoforms (Bruce and Margolis, 2002; Lai et al., 2001; Schroeder and Myers, 2008). The *FOXP2* gene shows a widespread pattern of expression across the majority of tissues and developmental time points. Nonetheless, within each tissue its expression appears to be tightly regulated in a complex pattern of expression with a high degree of conservation across species (Ferland et al., 2003; Lu et al., 2002; Schroeder and Myers, 2008; Shu et al., 2001, 2007).

The identification of *FOXP2* opened up a whole field of research encompassing a wide range of disciplines including neuroimaging, animal models (primarily mouse and songbird), molecular studies of gene function and expression, and population and evolutionary studies (reviewed in Fisher, 2006; Fisher and Scharff, 2009). Although the exact role of *FOXP2* in the cause of verbal dyspraxia has yet to be elucidated, it is clear that this gene is of particular importance in the development of brain regions responsible for fine motor control (motor cortex, striatum, and cerebellum) and that its disruption has exceptionally severe consequences for the development of speech. Many detailed reviews of *FOXP2* have been previously published and we refer the reader to these for a more in-depth discussion of this gene (Fisher, 2006; Fisher and Scharff, 2009).

Despite its obvious importance in severe and rare forms of verbal dyspraxia, it seems increasingly unlikely that *FOXP2* represents a general risk factor for genetically complex forms of language impairment (Meaburn et al., 2002; Newbury et al., 2002; O'Brien et al., 2003), nor is it expected to play a role in the related disorder autism (Gauthier et al., 2003; Laroche et al., 2008; Li et al., 2005; Marui et al., 2005; Wassink et al., 2002). However, the identification of this gene heralded a new era for the genetic study of speech and language as it allowed the identification of some of the biological pathways and mechanisms important for speech and language acquisition processes. Since *FOXP2* is a transcription factor, it regulates the expression of other genes, some of which may be expected to be involved in more common forms of speech and language deficits. Gene-targeting screens indicate that the FOXP2 protein is responsible for the downregulation of between 300 and 400 neural genes, many of which functionally represent good candidate genes for speech and language disorders (Spiteri et al., 2007; Vernes et al., 2007).

#### CNTNAP2

The list of genes regulated by FOXP2 includes a gene on chromosome 7 known as *CNTNAP2* (OMIM 604569) (Spiteri et al., 2007; Vernes et al., 2007). Recent evidence indicates that this gene may play a role in susceptibility to genetically complex forms of language impairment (Vernes et al., 2008). This study found that nine common genetic variants, involving changes at single base pairs (single-nucleotide polymorphisms) across the *CNTNAP2* sequence, were significantly correlated (minP = 5.0 × 10^−5^) with reduced performance across a number of linguistic measures (expressive and receptive language and phonological short-term memory) in a cohort of language-impaired families (Vernes et al., 2008). The mechanism by which these SNPs might alter *CNTNAP2* function has yet to be elucidated, and these findings have yet to be replicated in additional language-impaired samples. However, alternative variations across this gene (including common variation and rare disruptions or mutations) have also been implicated in a range of neurodevelopmental disorders including autism (Alarcón et al., 2008; Arking et al., 2008; Bakkaloglu et al., 2008; Jackman et al., 2009; Poot et al., 2010; Rossi et al., 2008), Gilles de Tourette syndrome (Belloso et al., 2007; Verkerk et al., 2003), schizophrenia (Friedman et al., 2008; O'Dushlaine et al., 2010), epilepsy (Friedman et al., 2008; Mefford et al., 2010; Strauss et al., 2006), ADHD (Elia et al., 2010), and learning disability (Zweier et al., 2009). Furthermore, *CNTNAP2* variation has also been associated with the normal personality trait “openness to experience” (Terracciano et al., 2010).

*CNTNAP2* encodes a neurexin protein that is responsible for the localization of potassium channels in developing neurons and plays an important role in the facilitation of axonal-glial interactions (Poliak et al., 2003; Zweier et al., 2009). Brain expression studies indicate that while this gene is evenly expressed across the rodent brain, it shows a specific pattern of expression in the song control nuclei of male songbirds (Panaitof et al., 2010) and is enriched in the frontal cortex of humans (Abrahams et al., 2007). Structural MRI studies of population cohorts found that individuals who carry two copies of the genetic “risk” variants previously associated with autistic disorder have significantly reduced volumes of gray and white matter across several brain regions, including the prefrontal cortex, fusiform gyri, occipital cortices, and cerebellum, which have previously been shown to be important in autistic disorder (Tan et al., 2010).

Thus current data suggest that *CNTNAP2* plays a fundamental role in neuronal development and that perturbations of its function may contribute to susceptibility to a diverse range of neurodevelopmental psychiatric disorders as well as normal variations in brain function (Corvin, 2010).

#### FOXP1

On the basis of *FOXP2* data, researchers have suggested that other forkhead binding genes represent good candidates for involvement in speech and language impairments. The human *FOX* gene family consists of over 40 members classified into 19 subfamilies (designated *FOXA* to *FOXS*) according to specific motifs within the DNA binding domain (Hannenhalli and Kaestner, 2009). The *FOXP* subfamily includes four genes (*FOXP1-4*) with diverse functions. The proteins encoded by these genes are found to bind to each other to form active heterodimer DNA binding molecules (Li et al., 2004, 2007). In particular, it has been suggested that FOXP1 and FOXP2 may have a particularly close relationship with overlapping functions that allow them to work in a cooperative manner during tissue development (Shu et al., 2007). In 2009, Pariani et al. described a patient with a deletion of chromosome 3 that disrupted the *FOXP1* gene and deleted three other transcripts (*EIF4E3*, *GPR27*, and *PROK2*). The primary clinical presentation was described as blepharophimosis (drooping eyelids) and arthrogryposis (contractures of hands and feet). However, the child also presented with developmental delays and speech and language deficits, which the authors hypothesized might be caused by the disruption of *FOXP1* (Pariani et al., 2009). A subsequent sequencing screen of the *FOXP1* coding sequence in 49 probands with a clinical diagnosis of developmental verbal dyspraxia described a nonsynonymous coding change that was observed in a single proband (Vernes et al., 2009). However, a similar change was also documented in an unaffected control individual (Vernes et al., 2009). More recently, a study described a patient with a Chiari I malformation (cerebellar tonsil abnormality) and epileptiform discharges with periods of motor arrest (Carr et al., 2010). This child was initially referred because of concerns over speech delay and was found to have a deletion involving only the *FOXP1* gene (Carr et al., 2010). Although the clinical presentation of this patient differed considerably from that observed by Pariani et al., the authors suggest that the blepharophimosis and arthrogryposis in the former case may be caused by the deletion of additional transcripts. They go on to surmise that the *FOXP1* disruptions are likely to account for the similarities in patient phenotype, namely deficits in motor development and speech delays. This hypothesis has recently gained support from a large-scale study for chromosome abnormalities in 1523 individuals with learning disability (Horn et al., 2010). This investigation identified deletions of the *FOXP1* gene in three unrelated patients (two males, one female) with moderate learning disabilities, global developmental delays, and severe speech and language disorders. MRI and electroencephalography of the patients did not reveal any gross structural brain abnormalities, and a similar chromosome deletion was again observed in a control individual who was not reported to have learning difficulties.

The above studies illustrate the difficulties of correlating genetic variation with specific phenotypic features. Even in cases where a sequence disruption can be definitively demonstrated across a number of individuals, the proof of causality is not always straightforward. While it is true that all of the above cases had severe speech and language difficulties, these impairments often formed part of a more global developmental delay that was not necessarily consistent across individuals. In addition, control individuals with no reported developmental problems were also found to carry disruptions of the *FOXP1* sequence. Furthermore, *FOXP1* has also been associated with the skin disorder vitiligo (Jin et al., 2010), host-response to hepatitis vaccines (Davila et al., 2010), and the likelihood of cancer survival (Fox et al., 2004; Jais et al., 2008). The diversity of these data are typical and by no means insinuate that these particular results must be spurious. Indeed, similar results have been described by the *CNTNAP2* and *FOXP2* studies described above—it is important to remember that no gene product acts in isolation. The function of genes may vary across different developmental time points and in different tissues and are modulated by a large number of stochastic variables that differ between individuals. Thus, it is unlikely that any genetic investigation will ever be able to draw a simple relationship between a given gene and a specific outcome. Furthermore, it is entirely possible that a transcription factor, such as FOXP1, which has the potential to intersect many biological pathways, may play a role in many seemingly unrelated disorders. Thus, given the recent convergence of evidence, it is likely that *FOXP1* is involved in biological processes that are particularly important in the development of speech and language.

The function of the FOXP1 protein in the brain remains unclear, but recent studies suggest that it may play a role in motor neuron diversification, through its interactions with Hox proteins (Dasen et al., 2008; Rousso et al., 2008); in neuronal migration, by gating Reelin signaling pathways (Palmesino et al., 2010); and in neuronal differentiation, via regulation of the Pitx3 protein (Konstantoulas et al., 2010).

Thus the accumulation of recent data suggests that, like its partner *FOXP2*, *FOXP1* may also be involved in the determination of neural circuitry important for the development of speech and language.

### Speech-Sound Disorder

Aside from *FOXP2*, its downstream targets, and its binding partners, researchers have reasoned that candidate genes or genetic regions implicated in dyslexia may also represent good candidates for speech and language disorders. This is particularly true for speech-sound disorder (SSD), which is characterized by the substitution or emission of speech sounds leading to intelligible speech. Speech-sound errors are a common feature of the language acquisition process but in children with SSD persist beyond the appropriate developmental time point. For example, it is estimated that while approximately 16% of 3-year-old children use inappropriate speech-sound formations, these errors only persist in 4% of children at 6 years of age (Shriberg et al., 1999). Speech-sound disorder is believed to be a specific problem with the development of phonological awareness (i.e., the conceptualization of speech-sound units) and, as such, of all the speech and language disorders is proposed to share the greatest etiological overlap with developmental dyslexia. In support of this theory, the presence of early speech problems has been shown to represent a significant risk factor for later literacy impairments, especially when the speech problems are accompanied by additional language-related deficits (Peterson et al., 2009; Raitano et al., 2004; Sices et al., 2007). These similarities have led researchers of SSD to focus entirely upon specific genetic regions that have previously been identified by linkage studies of dyslexia, namely chromosomes 1p, 3, 6p, and 15q (Scerri and Schulte-Körne, 2010), and in some cases, this approach has proved successful. Significant levels of linkage (minP = 0.00002) have been described between chromosome 3 (DYX5, OMIM 606896) and phonological memory and phonological decoding traits in SSD families (Stein et al., 2004). Suggestive levels of linkage have been reported on chromosomes 1 (DYX8, OMIM 608995) (minP = 0.0009, Miscimarra et al., 2007) 6 (DYX2, OMIM 600202) (minP = 0.0006, Smith et al., 2005), and 15 (DYX1, OMIM 127700) (minP = 0.004, Smith et al., 2005). An additional study found linkage to a binary affection status of SSD and quantitative measures of oral motor control, articulation, and phonological short-term memory in a region that flanked the DYX1 locus (Stein et al., 2006, 15 Mb distal). This region has previously been implicated in autism (AUTS4, OMIM 608636) and is commonly deleted in Prader-Willi Syndrome (Cook et al., 1998). It is possible that this result may represent variations in the precise positions of peaks of linkage, which may be caused by differences in genetic structure, or it may be that there are two loci on chromosome 15 that contribute to SSD etiology, one of which overlaps with the autism locus and a second of which coincides with DYX1.

Recent targeted association studies in dyslexic families have led to the identification of specific candidate genes within the dyslexia linkage loci mentioned above (reviewed in Scerri and Schulte-Körne, 2010). Intriguingly, many of these genes have functions in neuronal migration, a critical step in cortex development (reviewed by Gabel et al., 2010). These data complement the findings of classical postmortem neuroanatomical studies of dyslexic individuals (Galaburda et al., 1985) and suggest that this process may play a role in the etiology of reading disorders. The direct evaluation of these specific risk variants in SSD populations would categorically provide an answer to the question regarding etiological overlaps between these two disorders.

### Specific Language Impairment

In contrast to SSD, investigations of SLI have applied a genome-wide linkage approach to the identification of candidate genes or regions. SLI is diagnosed as an unexpected disorder in the acquisition of language despite adequate intelligence and opportunity and in the absence of any explanatory medical conditions (e.g., autism or hearing impairment) (Law et al., 2000). By definition, SLI is a very heterogeneous disorder that includes disorders of speech that have no obvious motoric etiology and incorporates deficits in other areas of expressive language (e.g., grammar, syntax, and semantics) as well as impairments in receptive linguistic abilities.

Two whole-genome linkage screens have been performed identifying three primary sites of linkage to SLI. These are on chromosome 13 (SLI3, OMIM 607134) (Bartlett et al., 2002), 16 (SLI1, OMIM 606711) (SLIC, 2002), and 19 (SLI2, OMIM 606712) (SLIC, 2002). These studies both involved relatively small numbers of affected families and yielded borderline significant p values. Nonetheless, of all the linkage data presented in this review, these three loci probably represent the strongest and most developed results as they have all been confirmed by subsequent replication and fine-mapping studies (Bartlett et al., 2004; Falcaro et al., 2008; Monaco, 2007; SLIC, 2004). The SLI1 region on chromosome 16 has been subject to the most in-depth investigation, and a targeted association study recently enabled the identification of specific genetic variants that may cause linkage to this locus (Newbury et al., 2009). Significant association was identified to two distinct clusters of common genetic variants located within the *ATP2C2* gene (OMIM 613082, minP = 2 × 10^−5^) and the *CMIP* gene (OMIM 610112, minP = 6 × 10^−7^). Variation in both of these genes was predominantly associated with performance on a task of phonological short-term memory, supporting the importance of memory processes in language acquisition (Newbury et al., 2009). Regression modeling indicated that *ATP2C2* and *CMIP* exerted an independent effect upon phonological memory ability, supporting a role for both regions in language ability. Within this targeted association study, a marginally significant, similar trend of association was observed to *ATP2C2* (minP = 0.006) in a sample of low-language performers selected from a population cohort. Thus, although this is perhaps the most thoroughly investigated locus described in this review, these results still require external replication, and a functional variant or mechanism has yet to be identified. Little is known regarding the functions of the CMIP and ATP2C2 proteins in the brain but both represent reasonable candidates. *ATP2C2* encodes a calcium ATPase responsible for the regulation of cellular calcium and manganese levels. Calcium is an important intracellular messenger and is involved in many neuronal processes including short-term memory (Dash et al., 2007). Manganese ions are toxic to neuronal cells at high concentrations (Perl and Olanow, 2007), and manganese deficiency is linked with epilepsy (Carl et al., 1986). *CMIP* was identified through a screen to identify large proteins expressed in the brain (Nagase et al., 2000) and encodes an adaptor protein thought to form part of the cytoskeleton (Grimbert et al., 2003). Cytoskeletal remodeling plays a key role in synaptic formation and neuronal migration (Heng et al., 2010). The CMIP protein has been shown to interact with FilaminA (Grimbert et al., 2004), mutation of which is associated with the neuronal migration disorder X-linked periventricular heterotopia (OMIM 300017). Other studies have found that it also interacts with the RelA (a subunit of the transcription factor NF-kappaB) (Kamal et al., 2009) and PI3 kinase proteins (Kamal et al., 2010), indicating that, as described for the FOX proteins above, CMIP may function across multiple biological pathways. Neither *CMIP* nor *ATP2C2* was identified as a FOXP2 target (Spiteri et al., 2007; Vernes et al., 2007), and no obvious relationships can be formulated between the two genes or with that of the *CNTNAP2* gene, indicating that the follow up of these data may implicate new pathways and processes that are important for the development of language. Interestingly, variants within *ATP2C2* have been associated with attention deficit hyperactivity disorder (ADHD, Lesch et al., 2008) indicating that the pleiotropic effects described for other language loci may also extend to this region.

In addition to the genome-wide approaches described above, one group has specifically investigated dyslexia-linked loci and the *FOXP2* region of chromosome 7 in language-impaired families (Rice et al., 2009). This study found a suggestive level of linkage to variants across all regions investigated on chromosomes 1 (DYX8), 3 (DYX5), 6 (DYX2), 7 (*FOXP2*), and 15 (DYX1), with various measures of linguistic ability. Association analyses of a denser panel of variants across *FOXP2* and *KIAA0319* revealed a marginal level of association (minP = 0.01) between *KIAA0319* and measures of speech, language, and reading and between *FOXP2* and reading, language, and articulation (Rice et al., 2009), indicating that the behavioral effects of these genes may extend across related phenotypes. This study further supports the etiological relationships between dyslexia and speech and language disorders, as suggested by the SSD investigations described above.

### Stuttering

The potential of founder and consanguineous pedigrees has recently been demonstrated by the identification of genetic pathways that may be important in the etiology of stuttering. Stuttering is defined as a disorder of speech fluency characterized by interruptions in speech flow and involuntary repetition or elongation of syllables (Prasse and Kikano, 2008). This disorder is relatively distinct from SSD and SLI and is persistent in approximately 20% of cases, particularly males (Prasse and Kikano, 2008). The majority of molecular genetic studies of stuttering have focused upon large consanguineous pedigrees in which stuttering appears to be familial and persistent. Four genome-wide linkage and one genome-wide association study have been performed for persistent familial stuttering. These investigations have yielded suggestive linkage to chromosomes 2, 3, 5, 7, 9, 12, 15, 18, and 21 and indicate a strong but complex sex effect in the relationships between risk variants (Raza et al., 2010; Riaz et al., 2005; Shugart et al., 2004; Suresh et al., 2006; Wittke-Thompson et al., 2007). Nonetheless, these studies yielded only moderate evidence of linkage or association to any one region, and the overlaps between the results were sparse. The most consistent and significant region implicated is on chromosome 12q (STUT2, OMIM 609261) (Riaz et al., 2005; Suresh et al., 2006). Sequencing of 45 genes across STUT2 revealed four coding mutations in the *GNPTAB* gene that were present in significantly more affected family members than unaffected individuals (Kang et al., 2010) The *GNPTAB* gene (OMIM 607840) encodes subunits of the N-acetylglucosamine-1-phosphotransferase enzyme, which plays a role in lysosomal targeting processes. An additional subunit of this enzyme is encoded by the *GNPTG* gene (OMIM 607838) on chromosome 16p and a downstream enzyme, N-acetylglucosamine-1-phosphodiester alpha-N-acetylglucosaminidase is encoded by the *NAGPA* gene on chromosome 16p (OMIM 607985). Sequencing of these related transcripts revealed additional coding mutations that were not observed in control individuals (Kang et al., 2010). Across the *GNPTAB*, *GNPTG*, and *NAGPA* genes, mutations were observed in 3.2% of the case chromosomes and 0.5% of the control chromosome screened (Kang et al., 2010). This study therefore implicates an additional biological mechanism in speech disorders—that of the lysosomal enzyme pathway. Mutations in the *GNPTAB* and *GNPTG* genes have previously been described to cause the metabolic disorders mucolipidosis types II and III, respectively (OMIM 252500 and 252605) (Paik et al., 2005; Raas-Rothschild et al., 2000). These disorders are not characterized by stuttering but instead by skeletal abnormalities, restricted joint movement, and disorders of the heart, liver, and spleen. In some cases (particularly type II), these features are accompanied by developmental delay and speech problems but not typically stuttering. However, none of the mutations identified by Kang et al. were identical to those that had previously been implicated in mucolipidosis. Furthermore, since mucolipidosis is a recessive disorder, affected individuals must carry two copies of a mutation. In the stuttering cohort, all but two individuals carried one normal copy of the *GNPTAB* and *GNPTG* gene. This may therefore account for the less severe phenotype described in this sample. This intriguing study has yet to be replicated or extended and there are many interesting questions that can be asked regarding the mechanism of susceptibility to stuttering in these individuals.

### Conclusions

In summary, the last decade has seen an explosion in our understanding of the genetic basis of speech and language disorders. The identification of *FOXP2* precipitated a whole field of research that continues to advance our understanding of the foundations of speech and language. It is likely that in the future, the investigation of the pathways that involve and intersect with *FOXP2* will identify many more candidate genes and mechanisms underlying speech and language disorders. The investigation of SSD has indicated that this disorder may share some genetic basis with developmental dyslexia. The study of SLI has enabled the identification of two candidate genes on chromosome 16 (*ATP2C2* and *CMIP*), and stuttering research has identified the lysosomal enzyme pathway (*GNPTAB*, *GNPTG*, and *NAGPA*) as another candidate mechanism.

Although the recent progress in this field is promising, it should be noted that, in comparison to other developmental disorders with a genetic contribution, speech and language disorders are relatively understudied. Many of the studies described above involved relatively small samples and have yet to be replicated in independent cohorts. As genetic technologies develop, the generation of larger data sets becomes progressively easier, allowing the identification of genetic variants with smaller effect sizes. Nonetheless, the application of these technologies demands the existence of large sample sets with consistent ascertainment and assessment standards. Even then, as we have seen above, and as evidenced by many other GWA studies, the interpretation of findings is far from straightforward. GWA studies in other complex disorders have enabled a revolution in candidate gene identification, but it is estimated that these loci still only account for a small proportion of the observed familial clustering. The source of this so-called “missing heritability” is still a matter of debate but has been suggested to arise from the presence of common genetic variation with minimal effect sizes, rare variants, structural deviations, and genetic modifications and interactions that are not well characterized by currently available GWA technology or algorithms (Manolio et al., 2009).

Thanks to advances in technology, however, alternative methodologies are now available to complement the GWA. It is now feasible to directly sequence entire genomes for increasingly large sample sizes, and this will be important in the identification of rare mutations (Cirulli and Goldstein, 2010). It is also now recognized that successful GWAs rely upon adequate sample sizes and precise phenotyping, and to achieve this, meta-analyses may be required. Improved sample sizes and the investigation of alternative populations both afford increased power to detect variants with smaller effect sizes and enable the investigation of gene-gene interaction effects (Manolio et al., 2009). In addition, the genome-wide investigation of epigenetic effects may identify alternative sources of genetic variation that are not readily captured by the GWA approach (Maunakea et al., 2010). It is envisaged that these advances will facilitate the identification of greater numbers of risk variants, and this is reflected in the current trend away from the identification of “disease genes” toward an understanding of the biological pathways that underlie specific pathologies. Given this appetite for ever-larger data sets, it is perhaps ironic that the gene that has provided us with the most information regarding speech and language disorders was identified by the investigation of a single family with a rare and specific form of speech impairment. The *FOXP2* story demonstrates the continuing validity of the genetic approach and exemplifies how such data can be used to complement and inform larger genome-wide and biological pathway investigations.

Through this review, we hope not only to have summarized the findings of genetic investigations of speech and language disorders but also to have indicated the challenges faced during the genetic investigation of such disorders. As genetic throughput expands, the true challenge is not the generation of data but the interpretation of the findings and the proof of causality. It will be interesting to see whether the recent findings pertaining to speech and language disorders can be replicated and how these avenues of investigation will eventually combine with those generated from larger high-throughput studies. Ultimately, it is hoped that the research summarized in this paper will allow a better understanding of the causes of speech and language disorders and the complex relationships between these impairments, thereby facilitating better diagnostic and treatment schedules for affected individuals.

## Figures and Tables

**Table 1:** Summary of Loci Implicated in Speech and Language Disorders

Chromosome Region	Gene	Disorder	Gene Identification Method	Identification References	Replication Notes
1p	NA	Speech-sound disorder	Targeted linkage	Miscimarra et al. (2007)	Region also linked to dyslexia
1q	NA	Stuttering	GWLA	Riaz et al. (2005)	
2q	NA	Stuttering	GWLA	Suresh et al. (2006)	Replicated by Wittke-Thompson et al. (2007)
3	NA	Speech-sound disorder	Targeted linkage	Stein et al. (2004)	Region also linked to dyslexia
3p14	*FOXP1*	Developmental delay with speech and language impairment	Translocation mapping and chromosome abnormality screen	Pariani et al. (2009); Carr et al. (2010); Horn et al. (2010); Vernes et al. (2009)	Replicated across several individuals with variable phenotypes
3q	NA	Stuttering	GWLA	Wittke-Thompson et al. (2007)	Linkage to 3q also found by Raza et al. (2010)
5q	NA	Stuttering	GWLA	Riaz et al. (2005)	Replicated by Wittke-Thompson et al. (2007)
6p	NA	Speech-sound disorder	Targeted linkage	Smith et al. (2005)	Region also linked to dyslexia
7q31	*FOXP2*	Verbal dyspraxia	GWLA with subsequent translocation mapping in an unrelated affected individual	Fisher et al. (1998); Lai et al. (2001)	Disrupted in a small no. of individuals with verbal dyspraxia. Not associated with SLI or autism.
7q	NA	Stuttering	GWLA	Riaz et al. (2005)	Replicated by Suresh et al. (2006) in male individuals only
7q36	*CNTNAP2*	SLI	Targeted association of candidate gene	Vernes et al. (2008)	Also associated with a range of other neurodevelopmental disorders. Association with SLI yet to be replicated.
9p	NA	Stuttering	GWLA	Suresh et al. (2006)	
12q23	*GNPTAB*	Stuttering	GWLA and subsequent targeted candidate gene sequencing	Kang et al. (2010); Riaz et al. (2005)	Linkage replicated by Suresh et al. (2006) when conditioning on chr 7q stuttering locus
13	NA	SLI	GWLA	Bartlett et al. (2002)	Replicated by Bartlett et al. (2004)
13q	NA	Stuttering	GWLA	Wittke-Thompson et al. (2007)	Overlaps with Bartlett linkage to SLI
15p	NA	Stuttering	GWLA	Suresh et al. (2006)	
15q	NA	Stuttering	GWLA	Wittke-Thompson et al. (2007)	
15q	NA	Speech-sound disorder	Targeted linkage	Smith et al. (2005)	Region also linked to dyslexia. Replicated by Stein et al. (2006)
16p13	*GNPTG*	Stuttering	Targeted candidate gene sequencing	Kang et al. (2010)	
16p13	*NAGPA*	Stuttering	Targeted candidate gene sequencing	Kang et al. (2010)	
16q24	*ATP2C2*	SLI	GWLA and subsequent targeted association	Newbury et al. (2009); SLIC (2002)	Linkage replicated by Falcaro et al. (2008); Monaco (2007); SLIC (2004). Association with SLI yet to be replicated. ATP2C2 associated with ADHD (Lesch et al., 2008)
16q24	*CMIP*	SLI	GWLA and subsequent targeted association	Newbury et al. (2009); SLIC (2002)	Linkage replicated by Falcaro et al. (2008); Monaco (2007); SLIC (2004). Association with SLI yet to be replicated.
18p	NA	Stuttering	GWLA	Shugart et al. (2004)	
19q13	NA	SLI	GWLA	SLIC (2002)	Linkage replicated by Falcaro et al. (2008); Monaco (2007); SLIC (2004).
21p	NA	Stuttering	GWLA	Suresh et al. (2006)	Linkage in female individuals only

GWLA, genome-wide linkage analysis; SLI, specific language impairment.
